# The effect of prolonged elbow pain and rTMS on TMS-evoked potentials: A TMS-EEG study

**DOI:** 10.1162/IMAG.a.7

**Published:** 2025-05-22

**Authors:** Nahian S. Chowdhury, Wei-Ju Chang, Donovan Cheng, Naveen Manivasagan, David A. Seminowicz, Siobhan M. Schabrun

**Affiliations:** Center for Pain IMPACT, Neuroscience Research Australia, Sydney, New South Wales, Australia; University of New South Wales, Sydney, New South Wales, Australia; Department of Medical Biophysics, Schulich School of Medicine & Dentistry, University of Western Ontario, London, Canada; The Gray Centre for Mobility and Activity, Parkwood Institute, St. Joseph’s Healthcare, London, Canada; School of Physical Therapy, University of Western Ontario, London, Canada

**Keywords:** rTMS, TMS-EEG, transcranial-magnetic stimulation, electroencephalography, pain, analgesia

## Abstract

Recent studies using combined transcranial magnetic stimulation (TMS) and electroencephalography (EEG) have shown that pain leads to an increase in the N45 peak of the TMS-evoked potential (TEP), potentially linked to changes in GABAergic activity. Conversely, 10 Hz repetitive TMS (10 Hz-rTMS), which provides pain relief, reduces the N45 peak. However, these studies used brief pain stimuli (lasting minutes), limiting their clinical relevance. The present study determined the effect of pain and 10 Hz-rTMS on the N45 peak in a prolonged pain model (lasting several days) induced by nerve growth factor (NGF) injection to the elbow muscle. In Experiment 1, TEPs were measured in 22 healthy participants on Day 0 (pre-NGF), Day 2 (peak pain), and Day 7 (pain recovery). In Experiment 2, we examined the effect of 5 days of active (n = 16) or sham (n = 16) rTMS to the left primary motor cortex (M1) on the N45 peak during prolonged NGF-induced pain, with TEPs measured on Day 0 and Day 4 (post-rTMS). Peak pain and muscle soreness was mild to moderate across experiments. In Experiment 1, there was no evidence for an increase in the N45 peak during prolonged pain. Exploratory analyses revealed evidence for a reduction in the N45 peak from Day 2 to 7, and a correlation between higher pain severity on Day 2 and a larger increase in the N45 peak. In Experiment 2, active rTMS reduced the N45 peak on Day 4 versus Day 0, with no effect in the sham group. Overall, our study showed that during prolonged pain, 5 days of 10 Hz rTMS induces a reduction in the TEP N45 peak. However, contrary to previous studies, prolonged pain itself did not increase the N45 peak. Taken together, this study provides weaker evidence for a link between the N45 peak and pain perception compared to previous research. Nonetheless, exploratory findings—such as a reduction in the N45 peak during the pain recovery phase and an individual-level relationship between increases in N45 and pain severity—suggest that further studies with larger sample sizes and more robust pain models are needed to clarify this connection.

## 1 Introduction

The perception of pain arises from a complex interplay between emotional, cognitive, and sensory mechanisms involved in the processing of afferent nociceptive inputs. Understanding these mechanisms is critical, as it can help us uncover pain biomarkers which can be applied to the diagnosis, prevention, and treatment of chronic pain (Apkarian, 2021;Marchand, 2021).

Recent years have seen the use of combined transcranial magnetic stimulation and electroencephalography (TMS-EEG) to identify novel brain markers linked to pain and analgesia (Chowdhury, Chiang, et al., 2023;Chowdhury, Millard, et al., 2024;De Martino et al., 2023;Ye et al., 2022). One of these markers is the TMS-evoked potential (TEP) N45 peak, which is usually observed ~45 ms after a TMS pulse is delivered to M1 (Farzan & Bortoletto, 2022). The amplitude of the N45 peak may reflect the activity of GABA_A_receptors, as it has been shown to respectively increase and decrease with GABA_A_receptor agonists and antagonists. While this relationship has not been replicated in recent research, it suggests that the N45 peak might serve as a potential, albeit not definitive, metric of cortical inhibitory processes (Darmani & Ziemann, 2019;Darmani et al., 2016;Premoli et al., 2014,^2018^). In one study on healthy participants, across three experiments, thermal heat pain lasting ~6 minutes was shown to induce an increase in the N45 peak relative to a pain-free baseline, with larger increases in the N45 peak associated with greater pain sensitivity (Chowdhury, Chiang, et al., 2023). In another study on healthy participants, a single session of repetitive TMS (rTMS) to the posterior-superior insular cortex delivered during a capsaicin heat pain model lasting 90 minutes was shown to decrease heat pain sensitivity, with these analgesic effects accompanied by a decrease in the N45 peak. Moreover, compared to sham rTMS, the reductions in pain intensity following active rTMS were not just associated with, but partially mediated by, reductions in the N45 peak (Chowdhury, Millard, et al., 2024). These findings suggest that short lasting pain is associated with an increase in the N45 peak, while rTMS-induced analgesia is associated with a decrease in the N45 peak, supporting a potential link between pain perception and GABA-mediated cortical inhibition.

While these preliminary data show promise for the use of the N45 peak as a potential pain biomarker, past studies used short-lasting painful stimuli in the minutes range, which may have limited clinical relevance. It is unknown whether similar results are observed with longer lasting pain, such as pain induced by intramuscular injections of nerve growth factor (NGF) (Hayashi et al., 2013;Schabrun et al., 2023). NGF injections induce prolonged musculoskeletal pain (Chowdhury, Chang, et al., 2022) that mimics the duration, time course, functional limitation, and hyperalgesia associated with chronic pain conditions (Bergin et al., 2015;Schabrun et al., 2023). A single NGF injection to the elbow muscle induces pain mimicking lateral epicondylalgia over several days, with pain peaking at 1–2 days post-injection, and pain resolving ~7 days post-injection (Bergin et al., 2015). Assessment of the effects of pain and rTMS on the TEPs during NGF-induced pain will, therefore, enhance our understanding of the cortical mechanisms implicated in pain and analgesia within a more clinically relevant prolonged pain experience.

The present study conducted two experiments which aimed to determine 1) the effect of prolonged lateral elbow pain induced by an NGF injection on the N45 peak and 2) the effect of 5 consecutive days of rTMS on the N45 peak within a model of prolonged lateral elbow pain. It was hypothesized that prolonged pain would increase the N45 peak relative to a pain-free baseline. Further, it was hypothesized that active rTMS would decrease the N45 peak relative to sham rTMS during pain.

## 2 Methods

### 2.1 Design

Both experiments were conducted at Neuroscience Research Australia Sydney. Experiment 1 was a longitudinal within-subjects design with participants followed for a period of 20 days. Experiment 2 was an assessor/participant blinded, randomized, sham-controlled, longitudinal, parallel design with participants followed for a period of 11 days. Experiment 2 was part of a larger study with three groups of participants: one group received individualized rTMS (where rTMS frequency was fixed to a person’s EEG peak alpha frequency), and the other two groups received either 10 Hz or sham rTMS. Outcomes collected before and after rTMS were: pain, TMS-evoked potentials, corticomotor excitability, and resting-state EEG measures. Experiment 2 of this paper only reports the data for the effects of 10 Hz and sham rTMS on TEPs, with the other outcomes to be reported elsewhere.

The primary outcome in both experiments was the TEP N45 peak, with other TEP peaks and pain being secondary outcomes. A detailed statistical analysis for the effects of rTMS on pain in Experiment 2 will be reported in a separate manuscript comparing individualized rTMS, 10 Hz rTMS, and sham rTMS. All procedures adhered to the Declaration of Helsinki, with written, informed consent obtained prior to study commencement. The study was approved by the local ethics committee at the University of New South Wales (HC230194 and HC230344).

### 2.2 Sample size calculation

A sample size calculation was conducted (G*Power 3.1.9.7) for each experiment based on available means/SDs reported in previous studies assessing the effect of acute pain on the TEP N45 peak (Chowdhury, Chiang, et al., 2023) and assessing the effect of 10 Hz rTMS on the N45 peak (Chowdhury, Millard, et al., 2024) during pain (seeSupplementary Materialfor G*Power output). In Experiment 1, for the effect of pain on the N45 peak (α = 0.05, β = 0.8, d = 0.75) a sample size of 16 was required to detect a within-group difference in the N45 peak between timepoints. We opted for a higher sample size of 22 participants to 1) increase statistical power to ~90%, therefore minimizing the risk of effect size underestimation based on a single study and 2) to allow for exploratory analysis of individual differences. For Experiment 2, for the effect of rTMS on the TEP N45 peak (α = 0.05, β = 0.8, effect size f = 0.31, correlation between repeated measures = 0.5) a sample size of at least 12 individuals was required to detect an interaction between time and group. We opted for a higher sample size of 16 participants in each group to increase power to ~90%, accounting for the risk of underestimating the true effect size based on a single study.

### 2.3 Participants

In Experiment 1, 22 healthy participants (11 females, 11 males, age: 22.0 ± 2.0) were recruited. In Experiment 2, 32 healthy participants (18 males, 14 females, age: 21.0 ± 2.0) were recruited. In both experiments, participants were included if they were between 18 and 65 years, and excluded if they presented with any acute pain; had a history or presence of chronic pain, neurological, musculoskeletal, psychiatric, or other major medical condition; were pregnant and/or lactating; or were contraindicated for TMS (e.g., metal implants in the head) as assessed using the Transcranial Magnetic Stimulation Adult Safety Screen questionnaire (Rossi et al., 2021).

### 2.4 Protocol

**Fig. 1.**
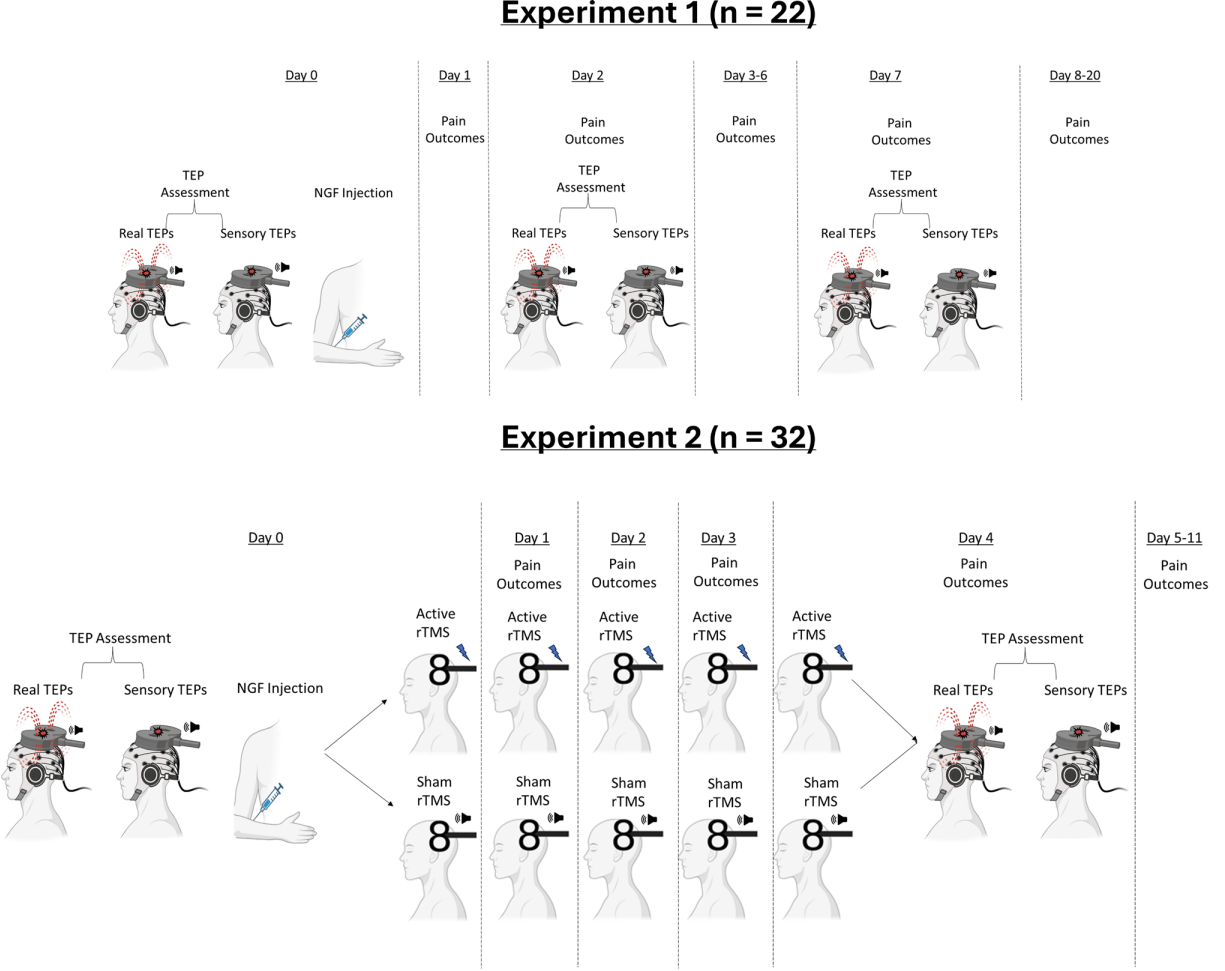
Diagram of the protocols in each experiment.

Figure 1shows the protocol for each experiment.

#### 2.4.1 Experiment 1

On Day 0, we first assessed “real” and “sensory” TEPs. Since the pulse intensity was set at subthreshold levels to avoid contamination from afferent feedback caused by muscle twitches, TEP assessment focused on the activation of the FDI, which typically has relatively lower resting motor thresholds (Kantak & Luchmee, 2020), reducing the likelihood of unintended activation in other muscles. Real TEP assessment involved TMS-EEG recording using a standard TMS coil. Sensory TEP assessment involved the use of a placebo coil which mimicked the auditory and somatosensory aspects of TMS, but without stimulating the brain. The rationale for using this coil is as follows: several studies have shown that a significant portion of TEPs do not reflect the direct cortical response to TMS, but rather auditory potentials elicited by the ‘clicking’ sound from the TMS coil, and somatosensory potentials elicited by the ‘flicking’ sensation on the skin of the scalp. This has led many authors to recommend the use of sensory control conditions to account for sensory contamination (Biabani et al., 2019;Chowdhury, Rogasch, et al., 2022;Conde et al., 2019;Rocchi et al., 2021). As such, sensory TEP assessment allowed us to establish whether any changes in the N45 peak were related to the direct cortical response to TMS, rather than the sensory response to TMS. TEP measurement on Day 0 was followed by an NGF injection to the right ECRB muscle to induce prolonged elbow pain for several days. Questionnaires assessing pain, functional impairment, and muscle soreness were sent to participant’s emails at 10 am and 7 pm each day from Days 1 to 20. TEPs were re-assessed on Day 2 (where pain was expected to peak) and Day 7 (where pain was expected to resolve) according to previous research (Bergin et al., 2015).

#### 2.4.2 Experiment 2

Prior to the study, each participant ID was randomly assigned to either an active or sham rTMS condition using a random number list generator (https://www.random.org/lists/). On Day 0, we assessed both real and sensory TEPs, followed by an NGF injection to the right ECRB. The allocations to either active or sham rTMS were then revealed to the experimenter (DC/NM) delivering rTMS via an automated email notification. The allocations were not revealed to the participant, or to the experimenters who collected TEP data or administered the injection of NGF (NC/WJC), and who performed data pre-processing and prepared the analysis plan (NC). Participants then received five daily sessions of 10 Hz active or sham (audible clicks) rTMS each day from Day 0–4 by the same experimenter. The final rTMS session on Day 4 was followed immediately by assessment of real and sensory TEPs. Pain was assessed in the lab on Days 0 to 4, and via electronic diaries sent at 10 am and 7 pm each day from Days 5 to 11. Participants were debriefed (experimenter WJC) after completing the study (contacted by phone or email) and their group allocation was notified. Group allocations were revealed to experimenter NC when the data collection, pre-processing, and analysis plan was complete.

### 2.5 Data collection procedures

#### 2.5.1 TEP assessment (both experiments)

Single, monophasic transcranial magnetic stimuli were delivered using a Duomag MP-EEG (Deymed, Czechia) and 70 mm figure-of-eight flat coil. EEG was recorded using a TMS-compatible amplifier (TruScan EEG 32, Deymed, Czechia) at a sampling rate of 3000 Hz. Signals were recorded from 32 passive electrodes, embedded in an elastic cap in line with the 10-5 system. Recordings were referenced online to ‘right mastoid’ and the ground electrode placed on the right cheekbone (Chowdhury, Millard, et al., 2024). Electrolyte gel was used to reduce electrode impedances below ~5 kΩ. In order to minimize the effect of the auditory response generated by the TMS coil click, the TMS adaptable auditory control (TAAC) masking toolbox (Russo et al., 2022) was used. Participants wore noise-cancelling headphones, and the sound was adjusted until they could barely perceive the single pulse at 90% RMT when applied to the stimulation site. In order to minimize the effect of the somatosensory response from the TMS coil, a thin layer of foam (5 mm) was placed between the TMS coil and the scalp.

Surface disposable silver/silver chloride adhesive electrodes were applied over the right first dorsal interosseous (FDI) muscle. The coil was oriented at 45° to the midline, inducing a current in the posterior-anterior direction. To identify the left M1 target, the scalp site (‘hotspot’) that evoked the largest motor evoked potential (MEP) measured at the first dorsal interosseous (FDI) was determined and marked. The rest motor threshold (RMT) was determined using the ML-PEST (maximum likelihood strategy using parametric estimation by sequential testing) algorithm (Awiszus & Borckardt, 2011) (target amplitude = 0.05 mV, step size = 6, difference threshold = 0.4 and initial intensity = 50). The test stimulus intensity was set at 90% RMT to minimize contamination of EEG signal from re-afferent muscle activation (Hernandez-Pavon et al., 2023). Although subthreshold intensity may produce a smaller N45 peak amplitude compared to suprathreshold stimulation (Ahn & Fröhlich, 2021), we have consistently observed this peak and its modulation by both pain and rTMS in previous studies using the same TMS protocol (e.g., target site, coil orientation) and pre-processing pipeline.

A real-time TEP visualization tool within the TruScan EEG software was used to confirm that artefacts (muscle, auditory) were minimal, and that, given the coil orientation and 90% RMT stimulus intensity, early peaks (<100 ms) at the stimulation site were evident (P30, N45, P60, N100) (Casarotto et al., 2022;De Martino et al., 2023;Hernandez-Pavon et al., 2023). If the N45 peak was undetectable due to channel noise, attempts were made to further reduce electrode impedance. Given the absence of established guidelines for sufficient amplitude or signal-to-noise ratio for the N45 peak, no strict quantitative threshold for these peaks was applied before commencing the test block. Real-time monitoring served solely as a visualization tool to confirm the presence of early peaks during setup and to assess and monitor signal noise within and between sessions. For each TEP measurement, 150 TMS pulses were delivered with a jitter of 1.75–2.25 ms. TEPs were collected with both standard figure-of-eight coil to assess real TEPs (DuoMag 70 BF Coil) and a placebo coil to assess sensory TEPs (Duomag 70 BF Placebo coil). The placebo coil mimicked the sensations of real TMS (auditory and somatosensory) without stimulating the area under the coil. The order of the real and sensory TEP assessment was randomized for each session. To evaluate participants’ perceptions of real and sensory TMS at the Day 0 session, participants were asked after each block of the real and sensory TEP assessments: “Do you believe you received real brain stimulation or sham brain stimulation?”

#### 2.5.2 Repetitive TMS (Experiment 2 only)

We used a rTMS protocol used in previous studies investigating the analgesic effects of rTMS (Seminowicz, de Martino, et al., 2018). For the active rTMS group, 10 Hz, biphasic TMS pulses were applied over the left primary motor cortex using a Magstim Super Rapid2 stimulator and a figure-of-eight coil. The location and intensity of rTMS was calibrated based on RMT of the right ECRB muscle (using the same hotspot and threshold procedures described previously). RMT was reassessed at each session to account for any changes in corticomotor excitability. During rTMS, the coil was held at 90˚ to the midline. 3000 stimuli (10 Hz, 30 trains of 10 seconds, 20-second intertrain interval) were delivered at 90% of RMT. For sham rTMS, a sham coil that looked identical to the active rTMS but produced only audible clicks was used to deliver the stimulation protocol identical to the one used for active rTMS.

#### 2.5.3 NGF injection (both experiments)

A sterile solution of recombinant human NGF (dose of 5 μg [0.2 ml]) was administered as a bolus injection into the muscle belly of the ECRB using a 1-ml syringe with a disposable needle (27-G), retracted ∼2 mm.

#### 2.5.4 Pain outcomes (both experiments)

For Experiment 1, we used the PRTEE (Rompe et al., 2007)Section 1and2. The PRTEE (Patient-Rated Tennis Elbow Evaluation) consists of two sections designed to assess pain and functional impairment.Section 1evaluates pain through five items, each rated on a scale from 0 (no pain) to 10 (worst imaginable pain). These items measure pain at rest, pain during repeated arm movements, pain while carrying a bag of groceries, pain at its least, and pain at its worst, with a maximum score of 50.Section 2assesses functional impairment through 10 items that focus on the difficulty experienced during specific tasks, such as turning a doorknob, carrying a bag, lifting a cup, opening a jar, pulling up pants, wringing out a towel, performing personal activities, household chores, employment-related tasks, and recreational activities. The maximum score for functional impairment is 100. In addition to the PRTEE, participants rated their muscle soreness on a scale from 0 to 6, with descriptive anchors for each level. A score of 0 indicated a complete absence of pain or soreness, while 6 represented severe muscle pain, soreness, stiffness, or weakness that limited movement. Intermediate scores captured varying degrees of pain or soreness, ranging from light pain felt only when touched to moderate or severe discomfort affecting movement or activity (Bergin et al., 2015;Hayashi et al., 2013). Questionnaires were administered via automated links sent to the participant’s email at 10 am and 7 pm each day. For Experiment 2, the PRTEESection 1was used to assess pain in session on Days 0 to 4, and via electronic diaries at 10 am and 7 pm each day from Days 5 to 11. In both experiments, any missing pain data was imputed using linear interpolation consistent with our previous work (Chowdhury, Taseen, et al., 2024).

### 2.6 Data processing

Pre-processing of the TEPs was completed using EEGLAB (Delorme & Makeig, 2004) and TESA (Rogasch et al., 2017) in MATLAB (R2021b, The Math works, USA), and based on previously described methods (Mutanen et al., 2020;Rogasch et al., 2017). First, the data were epoched 1000 ms before and after the TMS pulse, and baseline corrected between -1000 ms and -5 ms before the TMS pulse. The period between -5 ms and 15 ms after the TMS pulse was removed and interpolated by fitting a cubic function. Noisy epochs were identified via the EEGLAB auto-trial rejection function (Delorme et al., 2007) and then visually confirmed. The fastICA algorithm with auto-component rejection was used to remove eyeblink and muscle artefacts (Rogasch et al., 2017). The source-estimation noise-discarding (SOUND) algorithm was applied (Mutanen et al., 2020) to further suppress noise at each channel. The signal was then re-referenced (to average). A band-pass (1–100 Hz) and band-stop (48–52 Hz) Butterworth filter was then applied.

In line with previous studies investigating the effects of pain on TEPs (Chowdhury, Chiang, et al., 2023), and rTMS on TEPs during pain (Ye et al., 2022), the average TEP was extracted from a-priori frontocentral region of interest (ROI) (‘Fz’, ‘F3’, ‘F4’, ‘FC1’, ‘FC2’, ‘C3’, ‘Cz’, ‘C4’) investigated in previous studies (Chowdhury, Chiang, et al., 2023;Chowdhury, Millard, et al., 2024). The N45 TEP peak from this ROI was identified for each participant using the TESA peak function (Rogasch et al., 2017), with a predetermined window of interest (40–60 ms) chosen to account for variation between participants in the latency of the peaks. Refer to theSupplementary Methodsregarding analysis and results for the other peaks (N15, P30, P60, N100, P180).

### 2.7 Statistical analysis

Bayesian inference was used to analyze the data which considers the strength of the evidence for the alternative versus null hypothesis, using R (Version 4.4.1). Bayes factors were expressed as BF_10_values, where BF_10_’s of 1–3, 3–10, 10–30, 30–100, and >100 indicated ‘weak’, ‘moderate’, ‘strong’, ‘very strong’, and ‘extreme’ evidence for the alternative hypothesis, while BF_10_’s of 1/3–1, 1/10–1/3, 1/30–1/10,1/100–1/30, and <1/100 indicated ‘anecdotal’, ‘moderate’, ‘strong’, ‘very strong’, and ‘extreme’ evidence in favor of the null hypothesis (van Doorn et al., 2021). We used informed priors based on effect sizes from the posterior distributions from our previous studies. These priors are described in detail in theSupplementary Material.

Experiment 1 followed a 2 (time: Day 0, 2, and 7) x 2 (stimulation: real vs. sensory TEPs) repeated-measures design. Our analysis focused on the following questions.

1. Main effects of stimulation: Is there a difference in the N45 peak between real and sensory TEPs, averaged across days?
2. Main effects of time: Is there a difference in the N45 peak across days 0, 2, and 7, averaged across stimulation?
3. Overall interaction and interaction contrasts between time and stimulation: Are the changes in the N45 peak across all days and from Day 0 to Day 2, Day 0 to Day 7, and Day 2 to Day 7, different according to stimulation type (real vs. sensory)?
4. Day comparisons: Is there a difference in the N45 peak between Day 0 and Day 2, and between Day 2 and Day 7 (separately for real and sensory TEPs)?

Experiment 2 followed a 2 (group: active vs. sham) x 2 (time: Day 0, 4) x 2 (stimulation: real vs. sensory TEPs) mixed model design. Our analysis focused on the following questions:

1. 3-way interaction between group, time, and stimulation: Is the change in N45 peak following active versus sham rTMS stronger for real TEPs compared to sensory TEPs?
2. 2-way interaction between group and time for real TEPs alone: When analyzing real TEPs alone, is the change in the N45 peak from Day 0 to Day 4 stronger for active versus sham rTMS groups?
3. 2-way interaction between group and time for sensory TEPs alone: When analyzing sensory TEPs alone, is the change in N45 peak from Day 0 to Day 4 stronger for active versus sham rTMS groups?
4. Change in N45 peak for active rTMS group: Is there a change in the N45 peak from Day 0 to Day 4 for the active rTMS group (separately for real and sensory TEPs).
5. Change in N45 peak for sham rTMS group: Is there a change in the N45 peak from Day 0 to Day 4 for the sham rTMS group (separately for real and sensory TEPs)?

To address these questions, we used the brms package, which implements Bayesian generalized mixed linear models (GLMMs), using an adaptive Hamiltonian Monte-Carlo Markov-Chain Algorithm in Stan (Carpenter et al., 2017). For each effect described above, we constructed a reduced model, which only included random effects (both slopes and intercept), and a full model which included both fixed and random effects. This allowed us to compute a Bayes Factor which determined the extent to which the full model improves the fit of the data compared to the reduced model. The shape of the fitted data distribution for each model (e.g., gaussian vs. skew normal) was based on the shape of the observed data distribution. We ran four Markov chains simultaneously each for 8000 iterations with the first 2000 of iterations discarded as warm-up samples to adaptively tune the Monte-Carlo Markov-Chain sampler. Convergence of the models was checked by confirming R-hat was ~1.00 and model fit checked using posterior predictive checks to visually confirm that simulated distributions matched the observed data density plot.

### 2.7.1 Additional exploratory analysis Experiment 1

As there is now increasing emphasis placed on investigating the individual-level relationship between changes in cortical excitability and pain and not only the group-level effect (Chowdhury, Chang, et al., 2022;Seminowicz, Thapa, et al., 2018;Seminowicz et al., 2019;Summers et al., 2019,^2020^), in Experiment 1, we used Bayesian linear models to determine the relationship between change in the N45 peak on Day 2 and PRTEE, functional impairment, and muscle soreness, separately for real and sensory TEPs, while accounting for age as a covariate.

### 2.7.2 Additional exploratory analysis Experiment 2

We used Bayesian GLMMs with factors group (active vs. sham) and time (Day 0 to Day 11) to 1) determine whether there was a difference in PRTEE across time between active and sham rTMS groups (main effect of group), and 2) whether this difference varied across timepoints (group x time interaction).

## 3 Results

### 3.1 Experiment 1 results

All participants attended all three sessions with no missing data. The NGF injection led to no side effects. The mean (±SD) RMT on Day 0, Day 2, and Day 7 was 57.3 ± 7.7, 57.7 ± 8.2, and 56.2 ± 7.8 respectively. The median (Q1, Q3) pain diary adherence (proportion of completed diaries/total number of diaries) was 0.97 (0.87,0.97). The median (Q1, Q3) time adherence (minutes from designated timestamp of 7 pm or 10 am) was 74 (7, 426) minutes.

On Day 0, 32% (7/22) of participants classified both TEP assessments as real stimulation, 36% (8/22) misidentified real as sham and sensory as real stimulation, 23% (5/22) correctly distinguished between real and sham stimulation, and 9% (2/22) labeled both as sham stimulation. We conducted a McNemar’s test to test the null hypothesis that the proportion of discordant pairs was equal (believing the assessment of real TEPs was sham and the sensory TEP was real vs. the real TEP as real and sensory TEP as sham). There was no evidence (p = 0.58) that the proportion of discordant pairs were significantly different, suggesting participants were unable to distinguish between the real and sham conditions.

Grand-average TEPs and scalp topographies for Day 0, 2, and 7 for real and sensory TEPs are shown inFigures 2and3respectively.Figure 4shows the grand-averages for the frontocentral ROI at Day 0, 2, and 7 for real and sensory TEPs. There was extreme evidence that the N45 peak was higher for real versus sensory TEPs (BF_10_= 4.16 x 10^7^) averaged across time and very strong evidence for no change in the N45 peak across days (BF_10_= 0.02) averaged across stimulation. There was anecdotal to moderate evidence for no interaction between time and stimulation averaged across days (BF_10_= 0.24), and when comparing Day 0 and 2 (BF_10_= 0.46), Day 2 and 7 (BF_10_= 0.39), and Day 0 to 7 (BF_10_= 0.32). Exploratory analysis revealed that when analyzing the real TEPs alone, there was strong evidence for no difference in the N45 peak between Days 0 and Day 2 (BF_10_= 0.07), moderate evidence for no difference between Days 0 and 7 (BF_10_= 0.30), and strong evidence for a decrease in the N45 peak from Day 2 to Day 7 (BF_10_= 23.69). When analyzing the sensory TEPs alone, there was moderate evidence for no change in the N45 peak from Days 0 to Day 2 (BF_10_= 0.13) and strong evidence for no change from Days 0 to 7 (BF_10_= 0.1), and Day 2 to Day 7 (BF_10_= 0.06).

**Fig. 2.**
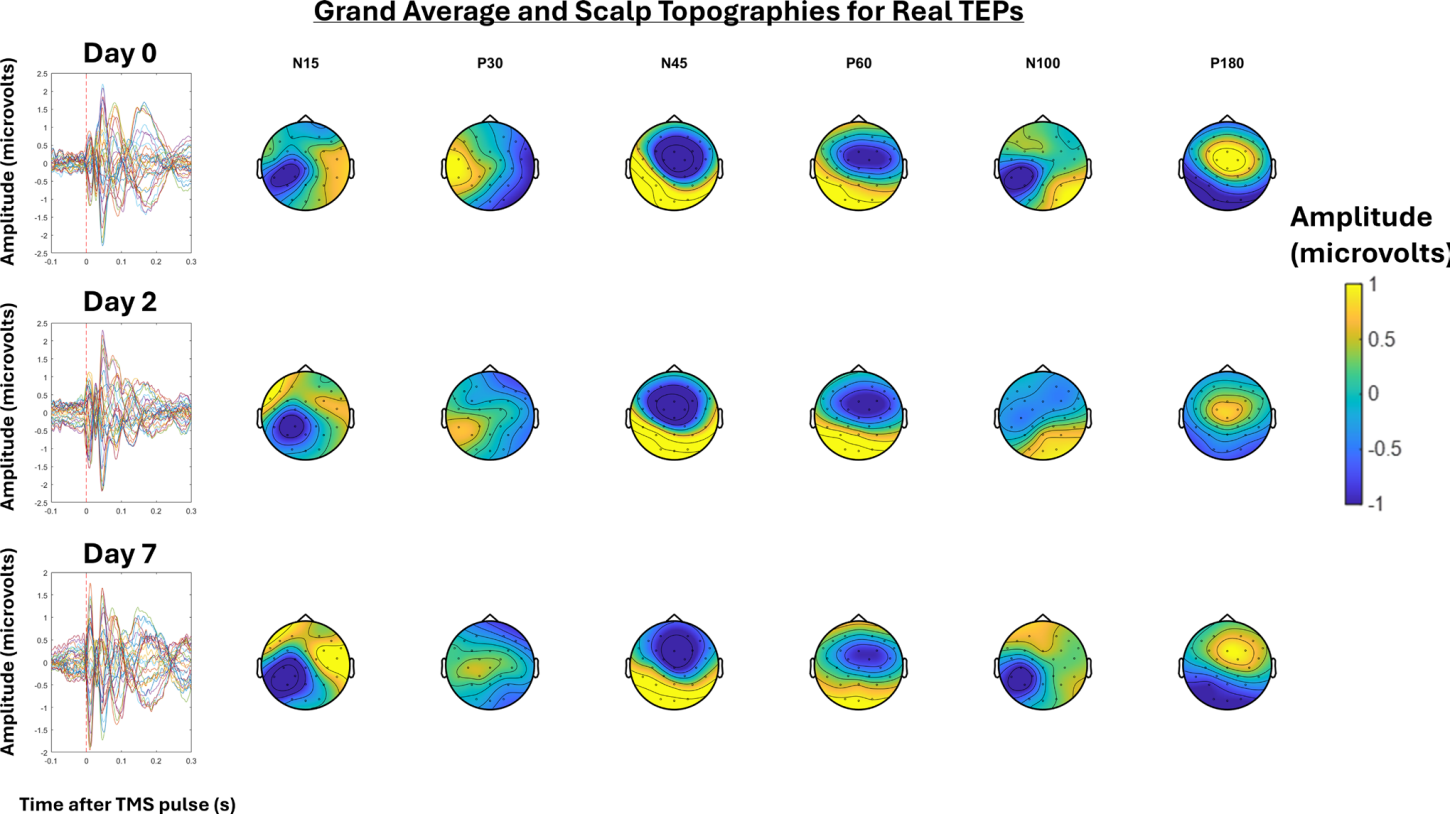
Left: Grand-average TEPs (n = 22) for all channels for the “real TEP” assessment at Day 0, 2, and 7. Right: Scalp topographies at timepoints where TEP peaks are commonly observed, including the N15, P30, N45, P60, N100, and P180.

**Fig. 3.**
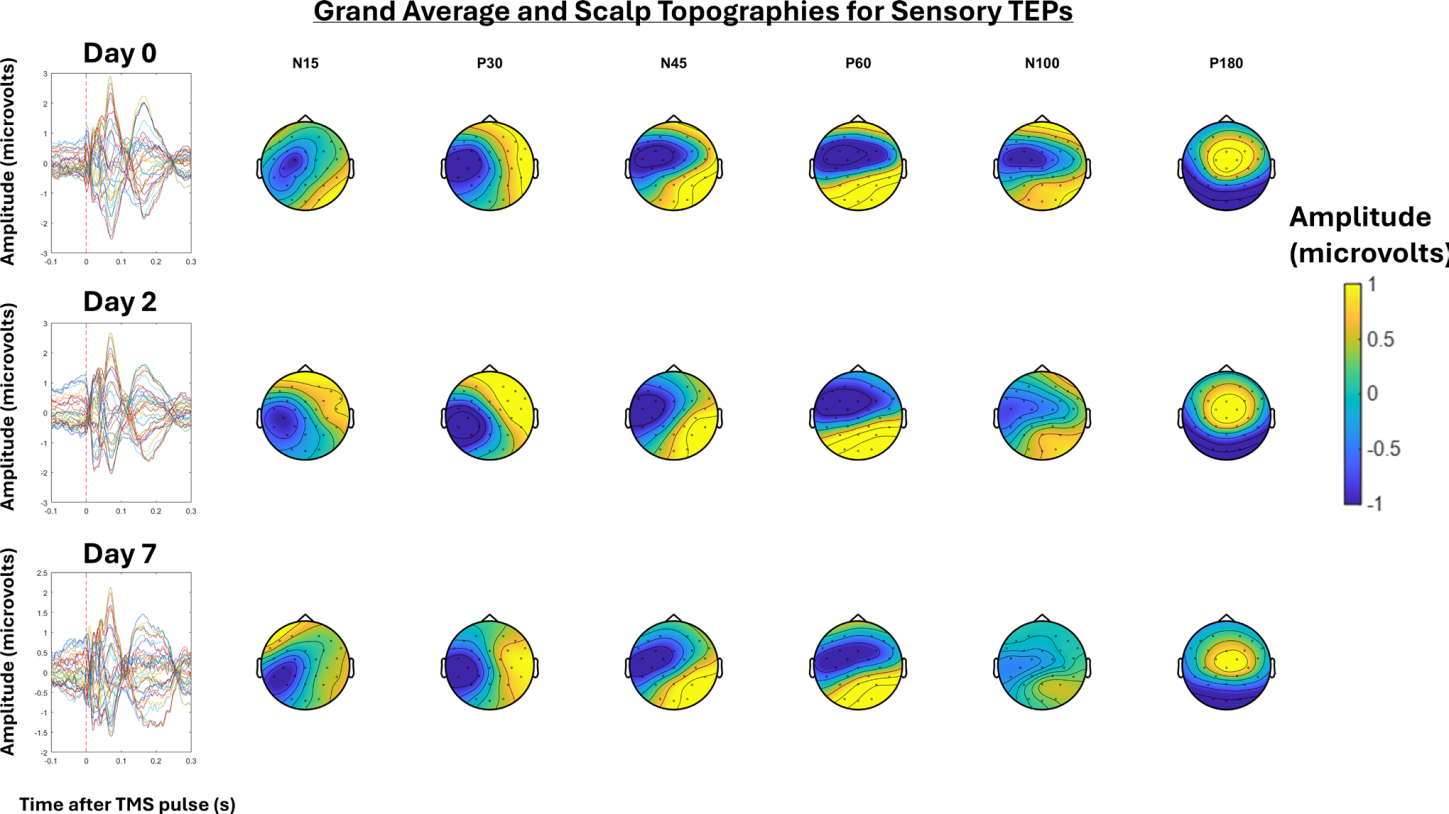
Left: Grand-average TEPs (n = 22) for all channels for the “sensory TEP” assessment at Day 0, 2, and 7. Right: Scalp topographies at timepoints where TEP peaks are commonly observed, including the N15, P30, N45, P60, N100, and P180.

**Fig. 4.**
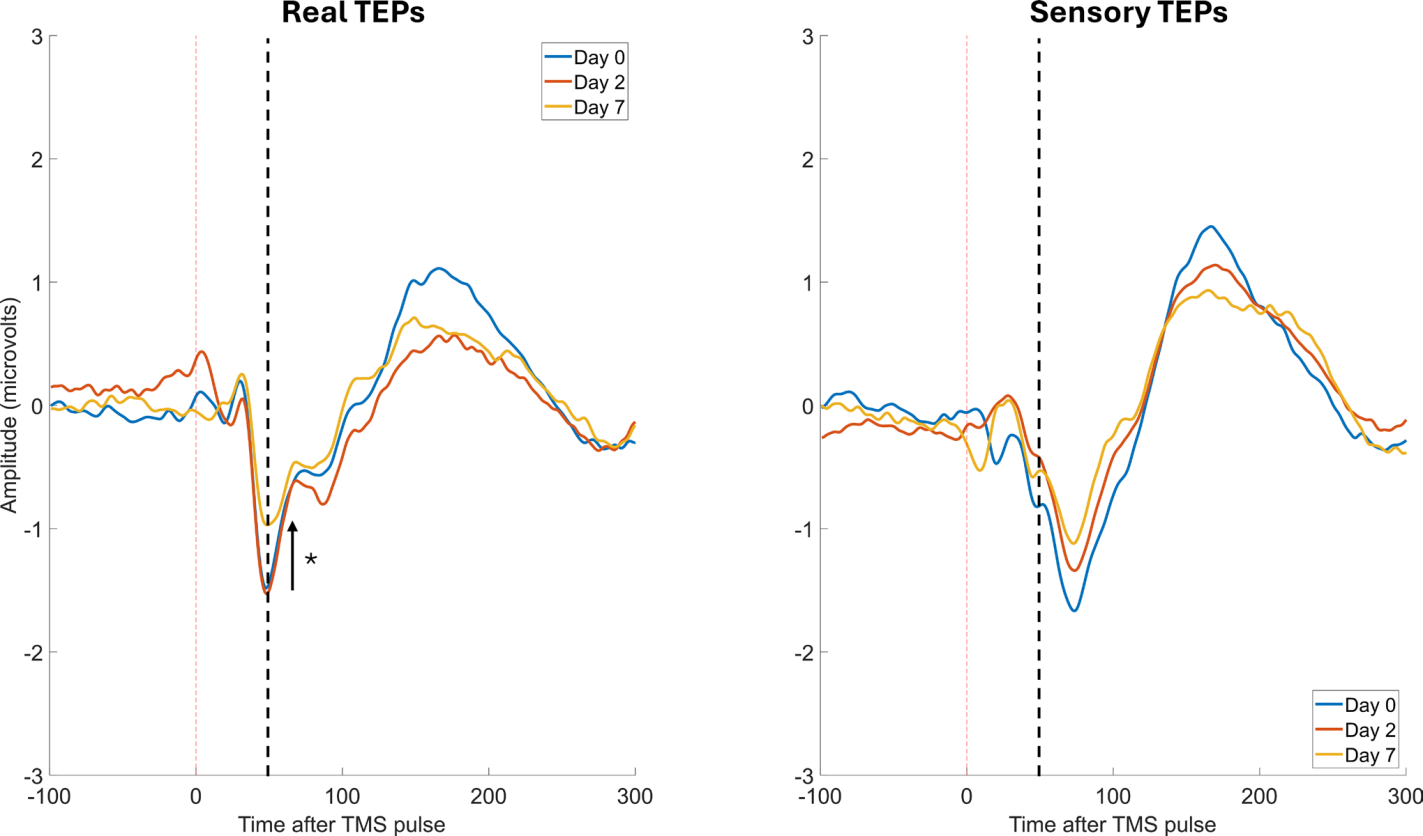
There was a reduction in the N45 peak from Day 2 to 7. The Grand-average TEPs (n = 22) for the frontocentral ROI for “real TEPs” assessment on the left and “sensory TEPs” assessment on the right, across Day 0, 2, and 7. The black dotted line shows the approximate timing of the N45 peak. The asterisk indicates evidence for a change.

**Fig. 5.**
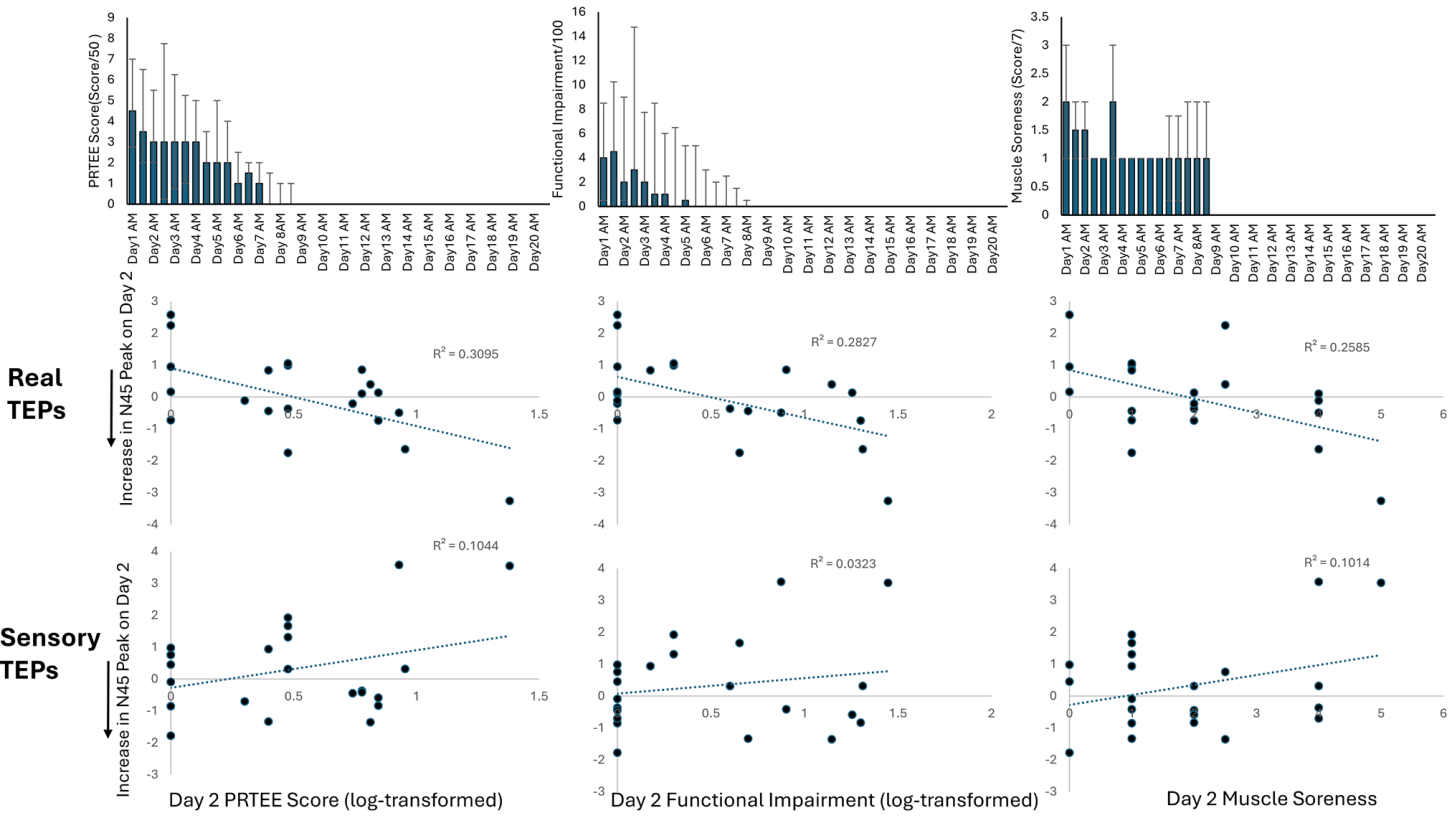
A larger increase in the TEP N45 peak was associated with higher pain, functional impairment, and muscle soreness on Day 2. Top panel shows the median (Q1, Q3) PRTEE, functional impairment, and muscle soreness scores across the 20 day period. The middle panel and lower panels show the relationship between the change in the TEP N45 peak (for real and sensory TEPs) at Day 2 and PRTEE score, functional impairment score, and muscle soreness score on Day 2 (averaged across am and pm timepoints).

Figure 5shows the median PRTEE and muscle soreness scores across Days 0–20. The median (Q1, Q3) maximum scores for PRTEE, pain at its worst, functional impairment, and muscle soreness were respectively 6/50 (3, 9.25), 2/10 (1,3), 6/100 (2, 14.75), and 3/7 (1,4). This indicates that, at its worst, participants experienced mild pain and functional impairment and moderate muscle soreness.Figure 5also shows the individual-level relationships between pain intensity, functional impairment, and muscle soreness on Day 2, and the increase in the N45 for both real and sensory TEPs measured on Day 2. For real TEPs, when controlling for age, a larger increase in the N45 peak from Day 0 to Day 2 predicted: higher pain with strong evidence (Spearman’s rho = -0.42, BF_10_= 12.96), higher functional impairment with moderate evidence (rho = -0.42, BF_10_= 6.82), and higher muscle soreness with moderate evidence (rho = -0.42, BF_10_= 4.71). For sensory TEPs, controlling for age, there was anecdotal evidence for no relationship between changes in the N45 peak and pain (rho = 0.16, BF_10_= 0.84), functional impairment (rho = 0.11, BF_10_= 0.51), and muscle soreness (r = 0.09, BF_10_= 0.95).

### 3.2 Experiment 2 results

All 32 participants attended the 5 lab sessions with no missing data. All participants tolerated the rTMS and NGF injection without side effects. The mean RMTs for the active rTMS group was 59.2 ± 6.0 and 59.3 ± 7.1 on Days 0 and 4 respectively. The mean RMTs for the sham rTMS group was 62.2 ± 7.9 and 63.9 ± 8.9 on Days 0 and 4 respectively. A Bayesian independent sample t-test showed anecdotal evidence for no difference in RMTs on Day 0 (BF_10_= 0.39) and Day 4 (BF_10_= 0.45). The median (Q1, Q3) pain diary adherence (proportion of completed diaries over total number of diaries) was 1 (0.92,1), and time adherence (minutes from designated timestamp of 7 pm or 10 am) was 61 (4, 146) minutes.

**Fig. 6.**
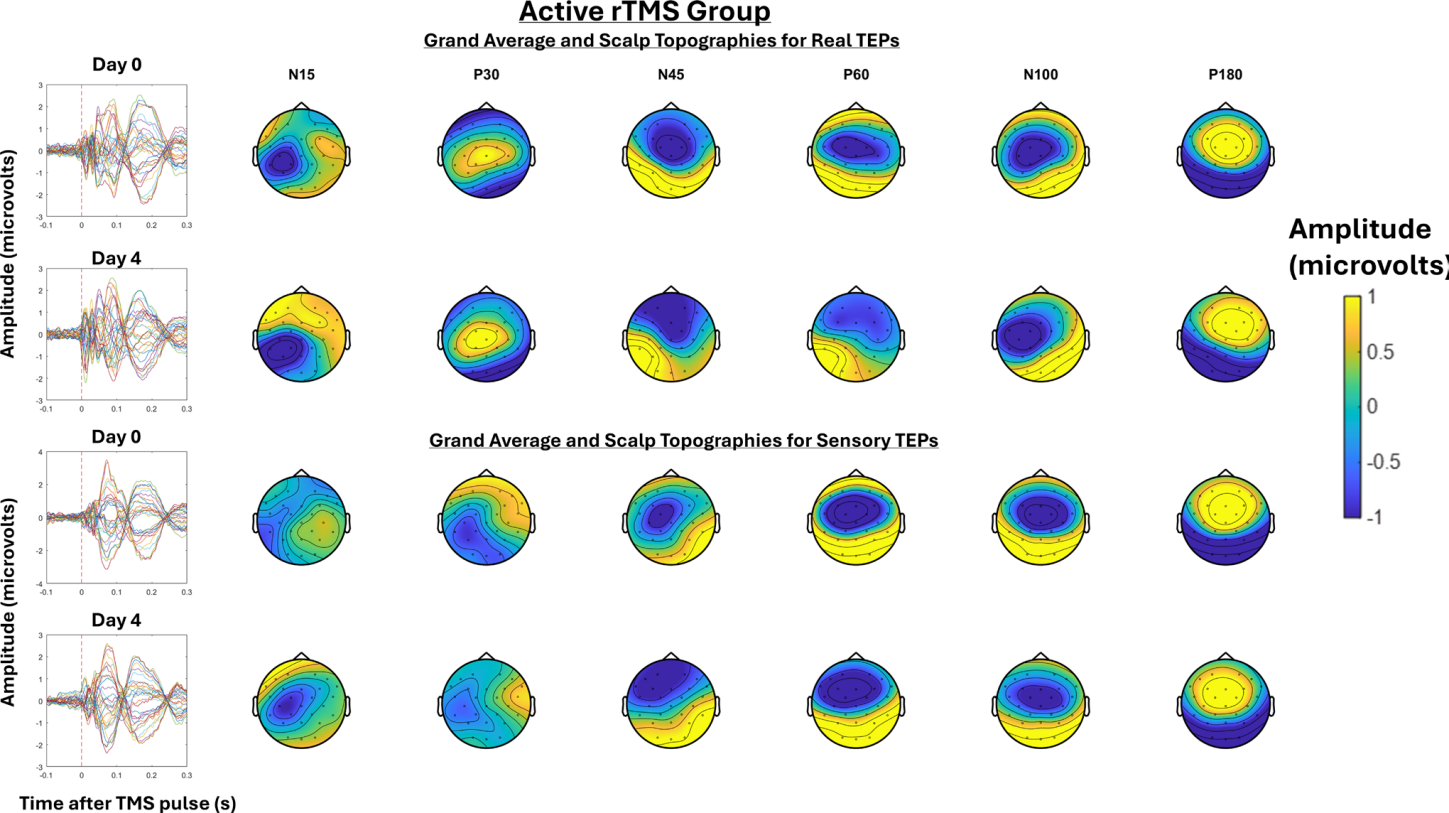
The left shows the Grand-average TEPs (n = 16) for all channels for the TEP assessment at Day 0 and 4 for the active rTMS group. The right shows the scalp topographies at timepoints where TEP peaks are commonly observed, including the N15, P30, N45, P60, N100, and P180. The top half shows the real TEP waveforms and scalp topographies, while the bottom half shows the sensory TEP waveforms and scalp topographies.

**Fig. 7.**
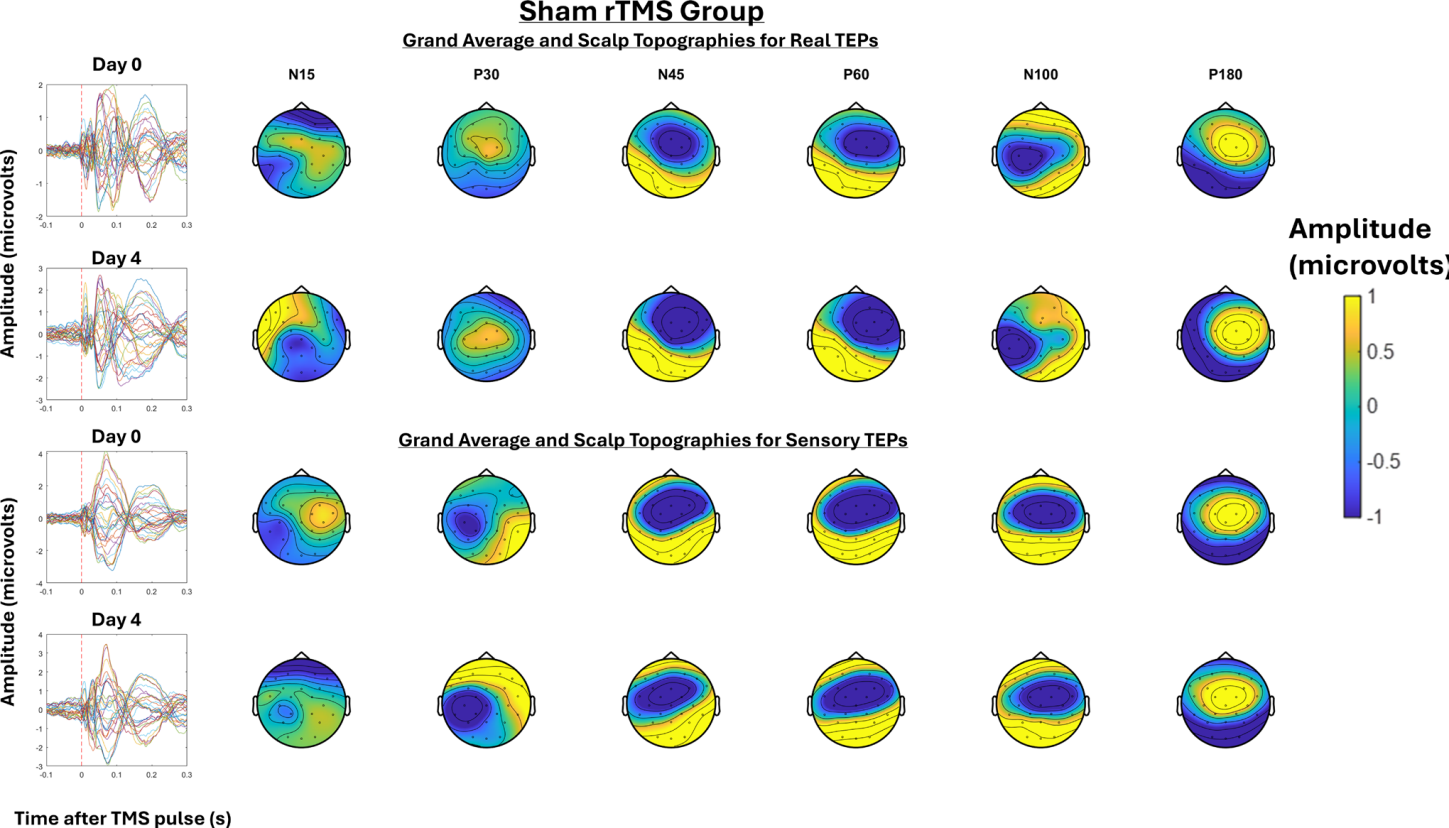
The left shows the Grand-average TEPs (n = 16) for all channels for the TEP assessment at Day 0 and 4 for the sham rTMS group. The right shows the scalp topographies at timepoints where TEP peaks are commonly observed, including the N15, P30, N45, P60, N100, and P180. The top half shows the real TEP waveforms and scalp topographies, while the bottom half shows the sensory TEP waveforms and scalp topographies.

**Fig. 8.**
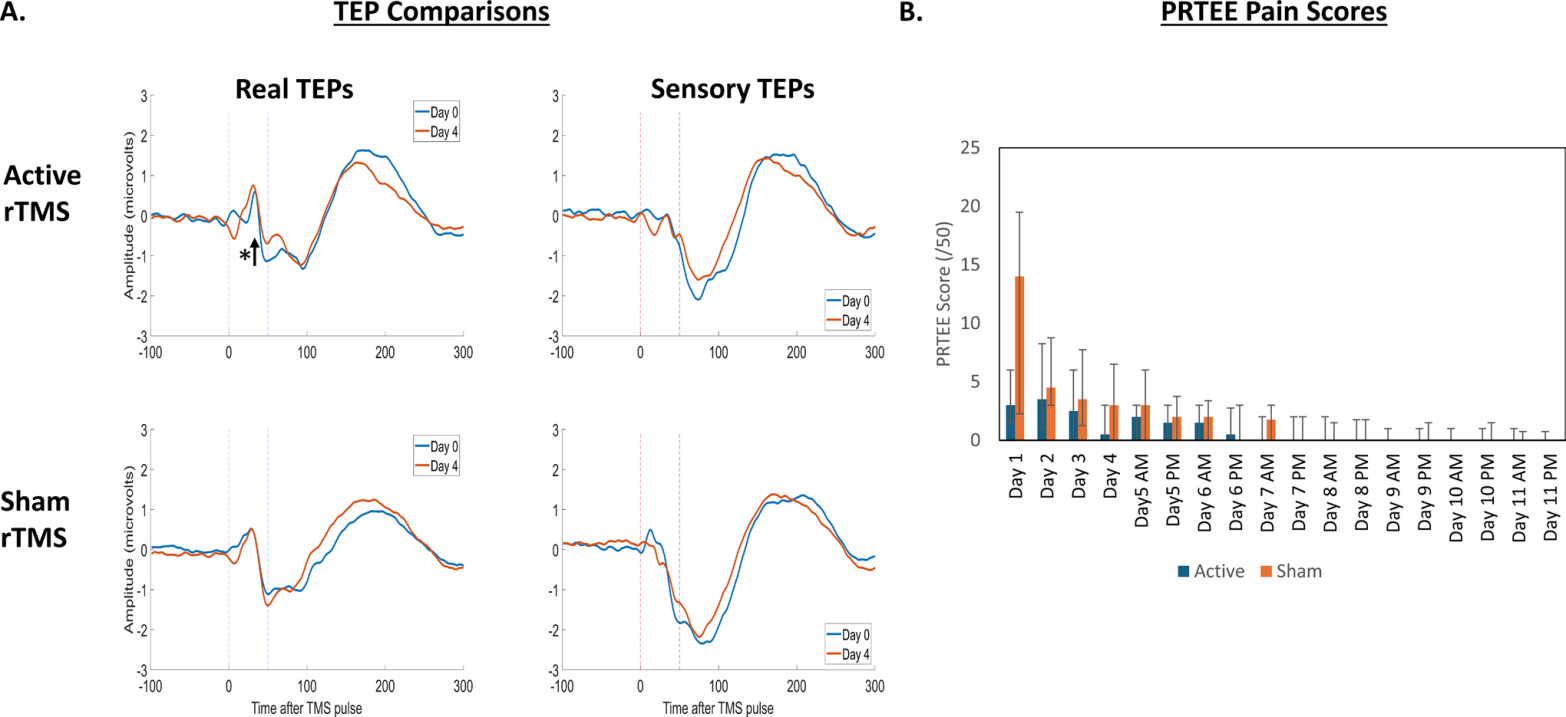
Five days of Active rTMS led to a decrease in the TEP N45 peak. (A) Grand-average TEPs for the frontocentral ROI for the active rTMS group (left, n = 16) and sham rTMS group (right, n = 16) on the right, with waveforms for real TEPs on the upper panel and waveforms for sensory TEPs on the lower panel. The blue dotted line shows the approximate timing of the N45 peak. The asterisk indicates Bayesian evidence for a change. (B) Median (Q1, Q3) PRTEE scores for the active and sham rTMS groups across the 11 day period.

Figure 6shows the grand-average TEPs and scalp topographies at Day 0 and Day 4 for the active rTMS group, for both real and sensory TEPs.Figure 7shows the grand-average TEPs and scalp topographies at Day 0 and Day 4 for the sham rTMS group, for both real and sensory TEPs.Figure 8Ashows the grand-average TEPs for the frontocentral ROI at Days 0 and 4 for real and sensory TEPs across active and sham rTMS groups. A Bayesian independent sample t-test revealed anecdotal evidence for no difference in the N45 peak between the active and sham rTMS groups on Day 0 for both real (BF_10_= 0.7) and sensory TEPs (BF_10_= 0.8). There was moderate evidence for a three-way interaction between stimulation, group, and time, such that, relative to sham, the decrease in the N45 peak following 5 days of active rTMS was stronger for real TEPs compared to sensory TEPs (BF_10_= 9.00). When analyzing real TEPs alone, there was moderate evidence that the decrease in the N45 peak was larger following 5 days of active rTMS compared to sham rTMS (BF_10_= 8.74). There was strong evidence that the N45 peak decreased from Day 0 to Day 4 in the active rTMS group (BF_10_= 12.33), and moderate evidence for no change in the N45 peak in the sham rTMS group (BF_10_= 0.17). For sensory TEPs, there was anecdotal evidence that relative to active rTMS, sham rTMS led to a larger decrease in the N45 peak (BF_10_= 2.62). There was anecdotal evidence that the N45 peak did not change from Day 0 to Day 4 in the active rTMS group (BF_10_= 0.12), and anecdotal evidence that the N45 peak decreased from Day 0 to Day 4 in the sham rTMS group (BF_10_= 0.88).

Figure 8Bshows the PRTEE scores for the active and sham rTMS group across Days 0–11. The median (Q1, Q3) maximum PRTEE was 9 (6, 15.5) for active rTMS group and 14 (5.5, 19.5) for the sham rTMS group. When analyzing the differences between groups using GLMM, there was moderate Bayesian evidence for no difference in PRTEE across days between active and sham rTMS groups (BF_10_= 0.25), and or for an interaction between group and time (BF_10_= 0.2).

## 4 Discussion

The present study aimed to determine the effects of pain and rTMS on the N45 peak within a model of prolonged lateral elbow pain induced by an NGF injection. In Experiment 1, contrary to our hypotheses, we did not find evidence for a group-level increase in the N45 peak during prolonged pain. However, exploratory findings showed strong evidence for a reduction in the N45 peak from Days 2 to 7, and that the increase in the N45 peak on Day 2 was associated with higher pain intensity. Experiment 2 showed that 5 days of 10 Hz rTMS over M1 led to a decrease in the N45 peak during prolonged lateral elbow pain, although there was no evidence for an analgesic effect of rTMS. These results are discussed in detail.

### 4.1 While there was no group-level evidence for an increase in the N45 peak, the individual-level correlations warrant further exploration

Experiment 1 examined the effect of prolonged pain on the N45 peak. Against predictions, prolonged pain did not induce an increase in the N45 peak when pain was at its peak. This is inconsistent with our previous work that showed that acute heat pain lasting several minutes led to an increase in the N45 peak (Chowdhury, Chiang, et al., 2023). One possible explanation for the lack of an effect is that pain and muscle soreness induced by the single injection to the ECRB was, on average, mild to moderate. Interestingly, however, exploratory analysis revealed evidence for a reduction in the N45 peak from Days 2 to 7, that is, during the pain recovery phase. While our design does not allow direct inference regarding the cause of this reduction, one possibility is that this reduction reflects an over-compensatory response during recovery from pain and muscle soreness, aligning with our previous findings that fluctuations in pain correlate with corresponding changes in the N45 peak (Chowdhury, Millard, et al., 2024). We encourage future studies to explore whether this reduction in the N45 peak during pain recovery is replicable and/or whether this reduction persists over a longer time period (i.e., beyond Day 7).

While we did not find group-level evidence for an increase in the N45 peak during pain, we found that an increase in the N45 peak was correlated with higher pain. While this should be interpreted with caution given the exploratory nature of the analysis, the finding is consistent with a previous study showing heat pain severity correlated with the increase in the N45 peak, supporting a potential link between pain and the N45 peak (Chowdhury, Chiang, et al., 2023). These findings also suggest that the NGF model is likely more suited for understanding individual differences in pain sensitivity, rather than the group effect of pain. This is consistent with literature exploring the effect of NGF pain on corticomotor excitability. In a systematic review (Chowdhury, Chang, et al., 2022), it was shown that prolonged pain (in the days-week range) did not decease corticomotor excitability on a group level, but that larger decreases in corticomotor excitability were correlated with higher pain severity. It is plausible that when pain lasts only seconds to minutes, this is an immediate threat to the affected area, resulting in more uniform changes in cortical excitability. However, when pain lasts for days or weeks, how each individual responds to pain and how brain regions involved in pain processing function vary. For example, in some individuals pain processing is more inhibited to prioritize task completion, while for others, pain processing dominates over other functions (Erpelding & Davis, 2013;Gombatto et al., 2015;Seminowicz et al., 2004). These differing pain adaptation strategies are associated with substantial variations in the brain’s response to pain over time (Chowdhury, Skippen, et al., 2023;Chowdhury et al., 2025;Seminowicz et al., 2019;Summers et al., 2019). Overall, our results suggest that the individual-level relationship between the N45 peak and pain warrants further exploration in larger samples.

### 4.2 During prolonged pain, 5 days of rTMS led to a reduction in the N45 peak

We found that 5 days of active rTMS decreased the N45 peak. These findings are consistent with our previous study showing that a single session of 10 Hz rTMS to the posterior superior insular cortex led to a reduction in the N45 peak during pain (Chowdhury, Millard, et al., 2024), suggesting that the effects of rTMS on the N45 peak generalized across different stimulation sites. These findings are also consistent with other research showing decreases in the N45 peak following multiple sessions of 10 Hz rTMS to the dorsolateral prefrontal cortex in patients with depressive symptoms (Voineskos et al., 2021). As we did not assess the N45 peak in response to pain alone (since the rTMS was given immediately after the NGF injection), it was not possible to determine whether any reductions in pain from pre to post-rTMS were correlated with, or mediated by, the reductions in the N45 peak induced by rTMS. As such, the functional relevance of the N45 peak reduction to pain relief cannot be directly inferred from Experiment 2. However, when considered alongside our prior work (Chowdhury, Chiang, et al., 2023;Chowdhury, Millard, et al., 2024)—where effects of pain on TEPs were replicated across three experiments in one study and where changes in the N45 peak mediated the analgesic effects of rTMS in another study—it seems reasonable to view these results as part of a broader body of evidence supporting a link between the N45 peak and pain. Overall, our findings suggest that rTMS can reduce the N45 peak in individuals experiencing prolonged experimental pain. We encourage larger-scale studies with TEP assessments at multiple timepoints to explore whether reductions in pain following rTMS are casually mediated by reductions in the N45 peak.

We did not observe a change in the N45 peak from Day 0 to Day 4 in the sham rTMS group, suggesting that prolonged pain over 4 days, without active intervention, does not increase the N45 peak. This contrasts with our previous study, where an increase in the N45 peak was observed following sham rTMS in a short-lasting pain model (Chowdhury, Millard, et al., 2024). As in Experiment 1, this discrepancy is likely related to the mild pain levels induced in the current model, which appeared even lower on Day 4 compared to Day 2—the time point when recordings were made in Experiment 1. As such, changes may have been present on Day 1, where pain was higher. We encourage future studies to, therefore, assess TEPs at more timepoints following NGF injections.

Note that there have been other recent studies (Tan et al., 2024;Ye et al., 2022) using TMS-EEG to explore the mechanisms of rTMS-induced analgesia. In these studies, 10 Hz rTMS was applied to either the motor cortex (Tan et al., 2024) or dlPFC (Ye et al., 2022), with TEPs and pain sensitivity to either cold pain (Ye et al., 2022) or capsaicin pain (Tan et al., 2024) assessed before and after rTMS. They reported that analgesia was associated with a reduction in the N100 peak, thought to reflect the combination sensory effects of TMS and GABA_B_ergic inhibition at the site of stimulation (Premoli et al., 2014). However, a key contrast to our study is that rTMS was not applied simultaneously with the pain model. Thus, the N45 and N100 may play different roles in pain perception during pain vs. outside of a painful experience. However, as the authors did not include sensory control conditions in their studies, another explanation could be that reductions in the N100 peak during these studies were associated with sensory aspects of TMS, particularly as our analysis (supplementary material) of the N100 suggests that sensory TEPs can also reduce over time. Therefore, further work is needed to disentangle the source of these changes.

### 4.3 Strengths, limitations, and future suggestions

A strength of the study is the use of a sensory control condition for the TEP measurements. Previous studies have shown that even when masking methods are used to minimize auditory or somatosensory effects of TMS, sensory contamination of TEPs is still present (Conde et al., 2019), as shown by strong temporal and spatial correlations in the signals of real TMS and sensory control conditions that mimic the auditory/somatosensory aspects of real TMS (Biabani et al., 2019;Chowdhury, Rogasch, et al., 2022). This has led to recommendations to use sensory control conditions (Biabani et al., 2019;Chowdhury, Rogasch, et al., 2022). However many authors opt not to use a sensory control condition due to the additional testing times and/or lack of resources to develop sensory control conditions (e.g., absence of a sham coil or sufficient apparatus to replicate auditory and somatosensory aspects of real TMS). Here, in both experiments, we included a sensory control condition using a coil (Duomag 70BF placebo coil) that could not only generate a click noise, but which could also induce a somatosensory response (flick sensation), which most commercially available sham coils are unable to achieve. We showed that the relationship between pain and the N45 peak occurred only for real and not sensory TEPs (Experiment 1), and that active rTMS only modulated real and not sensory TEPs (Experiment 2), thus strengthening our conclusions.

Despite the abovementioned strength, the main limitation across both experiments was the use of a single NGF injection, inducing a mild pain that may not have been sufficient to induce an effect on the N45 peak in Experiment 1, and may also have reduced the magnitude of any rTMS-induced analgesic effects in Experiment 2. Future research should consider using multiple NGF injections to induce pain with higher severity and longer duration. Another potential limitation of the study is the use of the marked hotspot method rather than neuronavigation. The latter has been shown to improve the consistency of coil positioning and orientation across sessions (Caulfield, et al., 2022). As such, the use of navigation-guided rTMS in the present study may have increased the reliability of TEP assessments and magnitude of the analgesic effects. Lastly, while the inclusion of a sensory control condition was a strength, the N100 peak of the sensory TEPs was quite large relative to real TEPs, possibly due to the louder click sound generated from the sham coil, even at the same stimulation intensity as the active coil (Takano et al., 2021). Future studies are encouraged to adjust for the sound intensity of the sham coil so that it matches the intensity in the active coil. In addition, the effectiveness of the sensory control condition could have been further evaluated by comparing specific aspects of the perception of the pulse (e.g., sharpness and focality) compared to the real condition.

## 5 Conclusion

Our study found that during prolonged experimental pain, five daily sessions of 10 Hz rTMS over M1 led to a reduction in the N45 peak. However, prolonged pain itself did not increase the N45 peak. Exploratory findings revealed a reduction in the N45 peak during the pain recovery stage and a positive association between higher pain severity on Day 2 and a larger increase in the N45 peak. Overall, while our findings provide some evidence for a link between the N45 peak and pain perception, this evidence is not as strong as in our previous studies. Nonetheless, the exploratory results highlight the need for further research with larger sample sizes and more robust pain models to better understand this relationship.

## Supplementary Material

Supplementary Material

## Data Availability

The data supporting the findings of this study are available from the corresponding author upon request due to the need for approval from the local ethics committee.
