# Supplementary material for: The effect of prolonged elbow pain and rTMS on TMS-evoked potentials: A TMS-EEG study
Source: Imaging Neurosci (Camb). 2025 May 22;3:IMAG.a.7. doi: 10.1162/IMAG.a.7 (PMC12319853; doi:10.1162/IMAG.a.7)
Supplement: Supplementary Material [file imag.a.7_supp.pdf]

Supplementary File for "The effect of prolonged elbow pain and rTMS on TMS-evoked Potentials: A TMS-EEG study"

Nahian S Chowdhury[1,2], Wei-Ju Chang[1,2], Donovan Cheng[1,2], Naveen Manivasagan[1,2], David A Seminowicz[3], Siobhan M Schabrun[4,5]

Affiliations

[1]Center for Pain IMPACT, Neuroscience Research Australia, Sydney, New South Wales, Australia, [2]University of New South Wales, Sydney, New South Wales, Australia, [3]Department of Medical Biophysics, Schulich School of Medicine & Dentistry, University of Western Ontario, London, Canada, [4]The Gray Centre for Mobility and Activity, Parkwood Institute, St. Joseph's Healthcare, London, Canada, [5]School of Physical Therapy, University of Western Ontario, London, Canada

Supplementary Methods

Sample Size Estimate Experiment 1

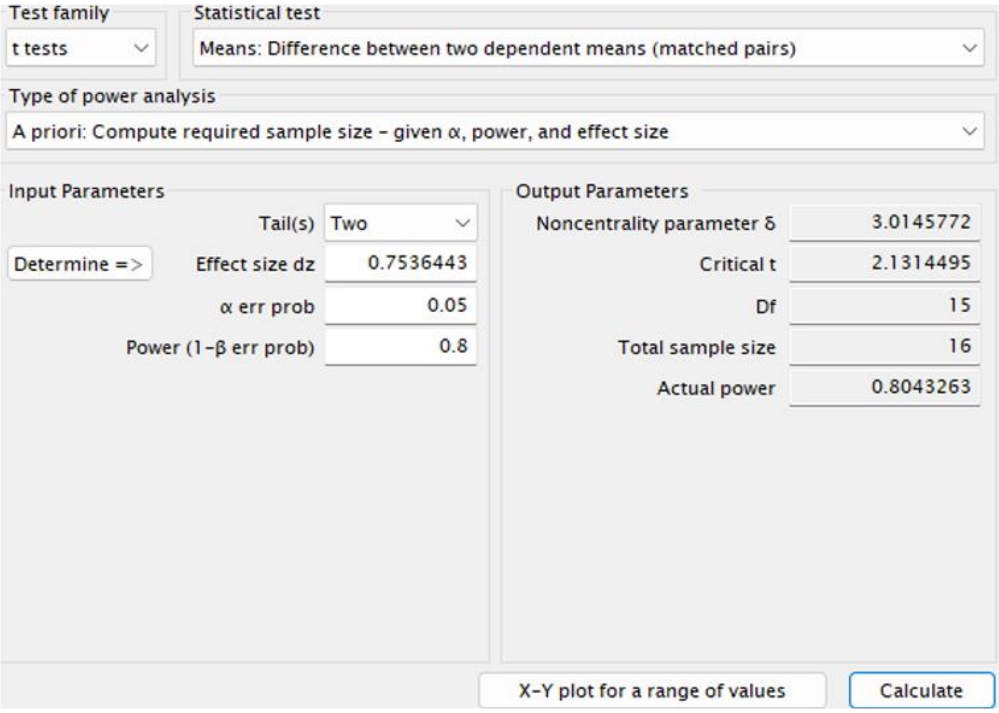

Sample Size Estimate Experiment 2

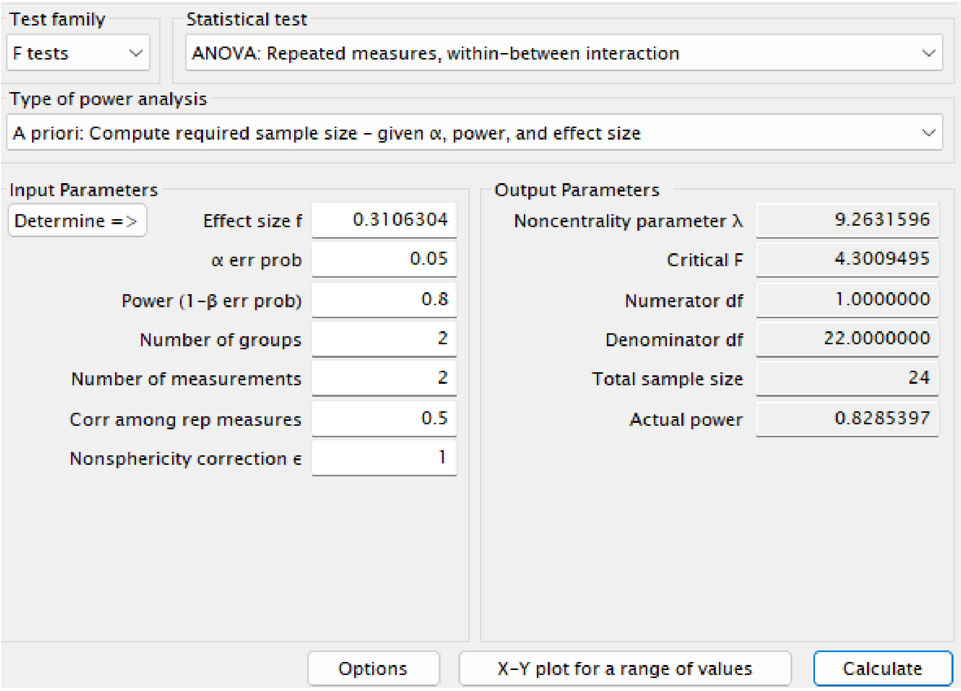

Bayesian priors

Priors were derived from the posterior distributions results of Bayesian GLMM from our previous studies that investigated similar effects (Chowdhury et al., 2023; Chowdhury et al., 2024). Standard deviations (SD) were selected based on the certainty of the effect size: where no prior information, was present, we used a normal distribution prior with a mean of 0 and SD of 1. Where existing prior information was present, we used the relevant posterior effect size along with an SD of 0.75, reflecting moderate uncertainty (since we only had one study to base the prior off).

Experiment 1. The prior data were sourced from a previous study (Chowdhury et al., 2023) with a similar design, including baseline, pain, pain recovery time points, and assessment of both real and sensory TEPs.

1. Interaction Between Time and Stimulation:

- Normal distribution (class = b) with a mean of 0.58 and SD of 0.75.

2. Main Effect of Stimulation:

- Normal distribution (class = b) with a mean of 2.37 and SD of 0.75.

3. Main Effects of Time:

- Normal distribution (class = b) with a mean of -0.18 and SD of 0.75.

4. Interaction Contrasts:

- Day 0 vs. Day 2: Normal distribution (class = b) with a mean of 0.76 and SD of 0.75.
- Day 2 vs. Day 7: Normal distribution (class = b) with a mean of 0.48 and SD of 0.75.
- Day 0 vs. Day 7: Normal distribution (class = b) with a mean of 0.58 and SD of 0.75.

5. Day Comparisons:

Real TEPs:

- Day 0 vs. Day 2: Normal distribution (class = b) with a mean of -1.02 and SD of .75.
- Day 2 vs. Day 7: Normal distribution (class = b) with a mean of +0.73 and SD of .75.
- Day 0 vs. Day 7: Normal distribution (class = b) with a mean of -0.32 and SD of .75.

Sensory TEPs:

- Day 0 vs. Day 2: Normal distribution (class = b) with a mean of 0 and SD of 0.75.
- Day 2 vs. Day 7: Normal distribution (class = b) with a mean of 0 and SD of 0.75.
- Day 0 vs. Day 7: Normal distribution (class = b) with a mean of 0.03 and SD of 0.75.

Experiment 2. The prior data were derived from a previous study (Chowdhury et al., 2024) with a design that included 2 time points (pre vs. post rTMS) and intervention (active vs. sham).

1. Three-Way Interaction Between Group, Time, and Stimulation

- This was weakly informed i.e. normal distribution (class = b) with a mean of 0 and SD of 1, since the interaction between group, time, and stimulation type has not yet been assessed.

2. Two-Way Interaction Between Group and Time for Real TEPs

- Normal distribution (class = b) with a mean of 0.48 and SD of 0.75.

3. Two-Way Interaction Between Group and Time for Sensory TEPs:

- Weakly informed: Normal distribution (class = b) with a mean of 0 and SD of 1 (since this effect has not been investigated before).

4. Change in N45 Peak for Active rTMS Group:

- Real TEPs: Prior was a normal distribution (class = b) with a mean of 0.44 and SD of 0.75.
- Sensory TEPs: Weakly Informed: Prior was a normal distribution (class = b) with a mean of 0 and SD of 1, as this has not yet been investigated.

5. Change in N45 Peak for Sham rTMS Group:

- Real TEPs: Prior was a normal distribution (class = b) with a mean of 0.35 and SD of 0.75.
- Sensory TEPs: Weakly informed: Prior was a normal distribution (class = b) with a mean of 0 and SD of 1.

Additional Analysis

Other peaks of the TEPs from the frontocentral ROI (N15, P30, P60, N100, P180) were identified for each participant using the TESA peak function, with predetermined windows of interest (N15: 12-20 ms, P30: 25-40 ms, P60: 55-70 ms, N100: 70-110 ms, P180: 150-200 ms). The same analysis approach (Bayesian GLMM) as with the N45 peak was

conducted on these other peaks. Priors for all analyses were a normal distribution with a mean of 0 and SD of 0.75, with the exception of the main effect of stimulation for Experiment 1. For this effect, the N15, P30 and P60 were expected to be larger for real TEPs than sensory TEPs (mean of +1, SD of 0.75), while the N100 and P180 were expected to be similar between real and sensory TEPs (mean of 0, SD of 0.75). This is based on evidence that the N100 and P180 largely reflect sensory aspects of TMS, while earlier peaks are less influenced by sensory contamination.

Supplementary Results

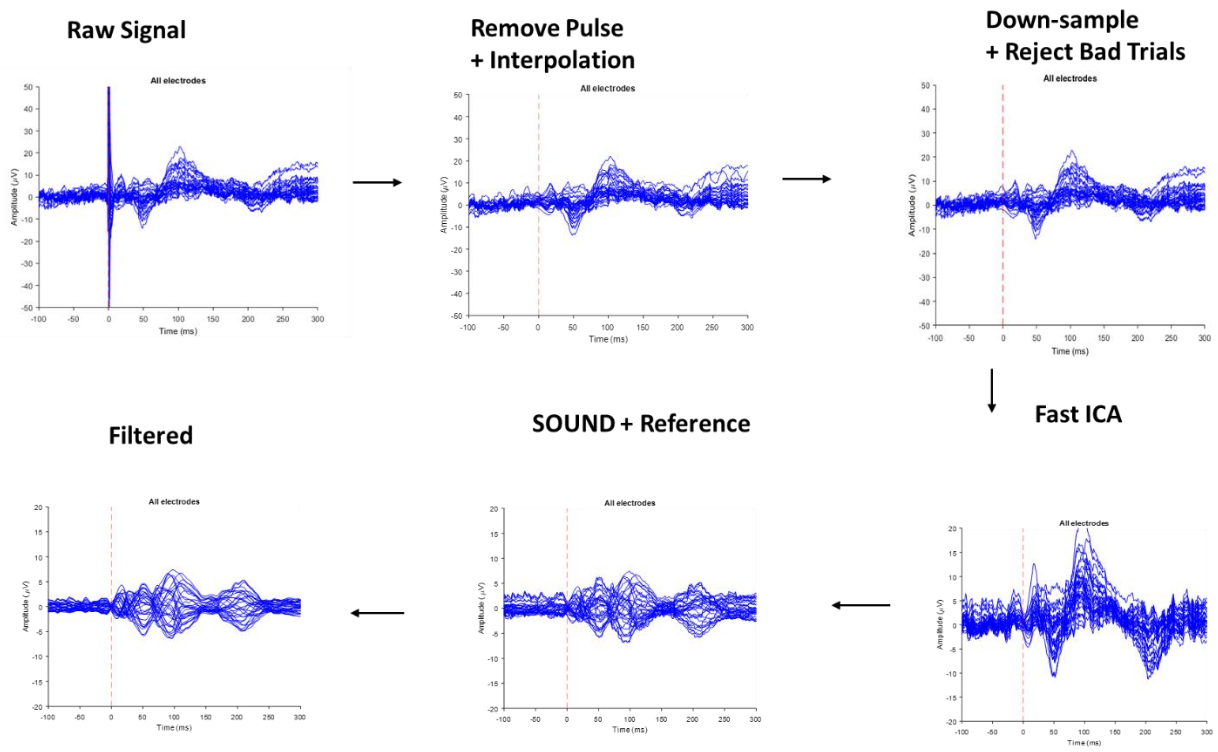

Supplementary Figure 1. Example Raw Data with Pipeline

Supplementary Table 1. Number of ICA components and bad trials excluded (Experiment 1).

		Day 0		Day 2		Day 7	
		Real	Sensory	Real	Sensory	Real	Sensory
**Components Excluded**	Mean	13.73	12.59	13.05	12.73	14.36	13.18
	SD	3.65	2.56	3.23	3.40	3.85	3.13
**Trials Excluded**	Mean	5.09	5.59	5.86	5.36	4.77	4.45
	SD	2.39	2.75	2.47	2.66	2.71	2.92

Supplementary Table 2. Bayes Factors which compare full vs. reduced models of the key effects (described in the Methods Section of the main paper), with dependent measure being the N15, P30, P60, N100 and P180 peaks (Experiment 1). There was moderate evidence that the P180 was larger in sensory TEPs vs. real TEPs, potentially due to the larger sound intensity generated by the the placebo coil.

	**N15**	**P30**	**P60**	**N100**	**P180**
**Time x Stimulation 2-way Interaction**	0.26	0.25	0.20	0.26	0.18
**Time Main Effect**	0.01	0.61	0.003	0.02	0.49
**Stimulation Main Effect**	0.03	0.01	0.005	2.19	**7.30**
**Interaction Contrast Day 0 vs Day 2**	0.83	0.55	0.57	0.50	0.33
**Interaction Contrast Day 2 vs Day 7**	0.52	0.51	0.49	0.43	0.59
**Interaction Contrast Day 0 vs Day 7**	0.48	0.52	0.42	0.45	0.31
**Real TEPs: Day 0 vs. Day 2**	0.03	0.05	0.05	0.05	0.34
**Real TEPs: Day 0 vs. Day 7**	0.02	0.05	0.04	0.05	0.36
**Real TEPs: Day 2 vs. Day 7**	0.04	0.03	0.04	0.08	0.02
**Sensory TEPs: Day 0 vs. Day 2**	0.23	0.24	0.03	0.28	0.33
**Sensory TEPs: Day 0 vs. Day 7**	0.09	0.30	0.03	0.33	0.82
**Sensory TEPs: Day 2 vs. Day 7**	0.14	0.14	0.06	0.10	0.16

Supplementary Table 3. Number of ICA components and bad trials excluded (Experiment 2).

			**Day 0**		**Day 4**	
			Real	Sensory	Real	Sensory
**Active rTMS**	Components Excluded	Mean	13.19	13.00	12.44	12.13
		SD	4.16	4.72	4.19	2.03
	Trials Excluded	Mean	5.25	5.19	4.88	4.88
		SD	1.44	2.61	2.28	2.28
**Sham rTMS**	Components Excluded	Mean	12.00	11.50	13.13	11.69
		SD	2.94	3.10	2.78	3.31
	Trials Excluded	Mean	5.06	4.38	5.25	4.81
		SD	2.52	2.66	2.82	2.34

Supplementary Table 4. Bayes Factors which compare full vs. reduced models of the key effects (described in the Methods Section of the main paper), with dependent measure being the N15, P30, P60, N100 and P180 peaks (Experiment 2). There was moderate evidence that rTMS modulated the P60 and P180 of real TEPs (for the P60 this modulation was stronger in real TEPs than for sensory TEPs as evident in the 3-way interaction). There was moderate evidence that the N100 component of sensory TEPs reduced from Day 0 to Day 4 following sham rTMS. The difference in this reduction relative to active rTMS was larger for sensory TEPs than real TEPs as evident in the 3-way interaction. This would suggest that sensory aspects of TEPs, in the absence of an active rTMS intervention, are prone to change over time.

	**N15**	**P30**	**P60**	**N100**	**P180**
**Group x Time x Stimulation 3-way Interaction**	1.92	0.33	**4.36**	**4.27**	1.08
**Real TEPs: Group x Time Interaction**	2.39	0.15	**3.36**	1.42	**4.96**
**Real TEPs: Day 0 vs. Day 4 Active rTMS**	0.03	0.13	0.11	0.92	2.18
**Real TEPs: Day 0 vs. Day 4 Sham rTMS**	0.03	0.03	0.08	0.19	0.21
**Sensory TEPs: Group x Time Interaction**	0.42	1.93	1.27	1.55	2.46
**Sensory TEPs: Day 0 vs. Day 4 Active rTMS**	0.10	0.03	0.21	2.22	0.59
**Sensory TEPs: Day 0 vs. Day 4 Sham rTMS**	0.02	0.02	0.82	**4.99**	0.20

Exploring the Potential for Floor Effects in the N45 peak values

The size of the N45 peak has been shown to be influenced by the intensity of TMS, with suprathreshold protocols leading to larger peak amplitudes (Ahn & Fröhlich, 2021). As such, one concern is whether there were floor effects in the N45 peak given we used a subthreshold TMS protocol**.** To determine this, we collated the data across both experiments (since we expected N45 peak to increase in Experiment 1 and decrease in Experiment 2). A box plot of the distribution of the change in N45 scores from baseline shows that the change values are spread relatively evenly in both directions. This suggests there was enough room for the N45 room to “shift” from its baseline position. If anything, there is a slight tendency towards a reduction in these values eliminating the possibility of a floor effect. If a floor effect had been present, the distribution of change values would be biased towards there being an increase in the N45 peak, with little room to reduce in magnitude. As a further note, we observed strong evidence that the N45 reduced from Day 4 to Day 0, further supporting the idea that it is unlikely there was a floor effect in N45 values.

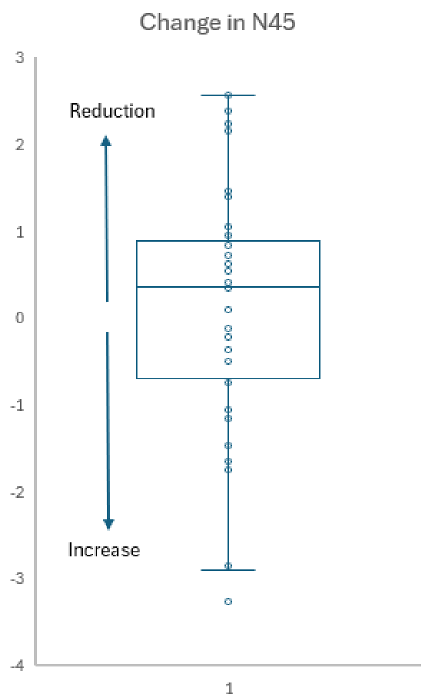

Supplementary Figure 2. N45 Change scores across Experiments.

References

Ahn, S., & Fröhlich, F. (2021). Pinging the brain with transcranial magnetic stimulation reveals cortical reactivity in time and space. *Brain Stimul*, *14*(2), 304-315.

Chowdhury, N. S., Chiang, A. K., Millard, S. K., Skippen, P., Chang, W.-J., Seminowicz, D. A., & Schabrun, S. M. (2023). Combined transcranial magnetic stimulation and electroencephalography reveals alterations in cortical excitability during pain. *Elife*, *12*, RP88567.

Chowdhury, N. S., Millard, S. K., De Martino, E., Larsen, D. B., Seminowicz, D. A., Schabrun, S. M., de Andrade, D. C., & Graven-Nielsen, T. (2024). Posterior-superior insula repetitive transcranial magnetic stimulation reduces experimental tonic pain and pain-related cortical inhibition in humans. *Pain*, 10.1097.
